# Psychiatric Disorders in Women Seeking Fertility Treatments: A Clinical Investigation in India

**DOI:** 10.22074/ijfs.2020.5759

**Published:** 2020-03-05

**Authors:** Ansha Patel, Podila Satya Venkata Narasimha Sharma, Pratap Kumar

**Affiliations:** 1 Department of Psychiatry, Kasturba Medical College, MAHE, Manipal, Karnataka, India; 2 Department of Obstetrics and Gynaecology, The Manipal Assisted Reproduction Centre, Kasturba Medical College, MAHE, Manipal, Karnataka, India

**Keywords:** Cross-Sectional Study, Distress, Infertility, Psychiatric Disorder, Women

## Abstract

Fertility treatments began in several countries, including India, in the1970s. Despite various advancements in intra uterine insemination (IUI) and *in vitro* fertilization (IVF), empirical investigations on the psychological endurance and emotional tolerance of Indian women to such treatments are rather scarce. Thus, the aim of this study is to estimate the prevalence of psychiatric disorders in Indian women seeking fertility treatments. It is a cross-sectional study with three hundred women participants undergoing various treatments at the Manipal Assisted Reproductive Centre, Kasturba Medical College, Karnataka, India. Psychiatric disorders were assessed in women using the “ICD- 10 Classification of Mental and Behavioural Disorders” followed by descriptive data analysis. The results show that 78% of women have psychological issues and 45% of them have a diagnosable psychiatric condition. Adjustment Disorders, Anxiety Disorders and Mixed Anxiety and Depression Disorder are established as the top three categories of diagnoses. The findings of this study suggest that women have a high emotional stake in infertility treatments. The data highlights the need for modification of the existing treatment protocol (in Indian clinics) in ways that ensure the emotional wellbeing of patients.

Globally, depression and anxiety are among the top causes for disease burden especially in middle-income countries such as India (1). WHO estimates for the period 2000-2015, show that together these psychiatric conditions contribute greatly to the total number of disability adjusted life years (1, 2), and their prevalence rates are estimated to rise by 2030 (3). These disorders are more common in women above the age of 18 years and in those with a co-morbid physical illness. Such ailments include hypertension, myocardial infarction, epilepsy, stroke, diabetes, cancer and tuberculosis, arthritis, chronic pain, back or neck problems, headaches, etc (4). However, their prevalence in those suffering from infertility is less documented.

Female infertility can be attributed to factors such as sexual dysfunctions, dyspareunia and vaginal causes, congenital defects in the genital tract, infections, chronic ill-health, cervical factors, uterine factors, tubal factors, ovarian factors, peritoneal and endocrinal factors (5). Psychiatric disorders in women undergoing infertility treatment are reported to be common (6). Review studies show that more than 50% of infertile patients face psychological problems. These are associated with variables like sex, number of cycles, type, length, costs of infertility evaluation and treatments (7-11). Other studies have revealed that a higher proportion of infertile women have psychiatric problems compared to fertile controls. Paranoid ideation, interpersonal sensitivity, and phobic anxiety are commonly found in childless women (9). Literature from the Indian context consists of studies that appear to have insufficient sample sizes and assessment biases. This literature indicates that both infertile men and women have subclinical as well as clinically significant psychiatric conditions (10). Evidenced based data emphasises that emotional distress in infertility makes a person vulnerable to complicated grief reactions, depression, dysthymia, reproductive mood disorders, anxiety disorders, adjustment disorders and sexual dysfunctions which compromise quality of life in men and women (11-20).The literature also suggests that there is an elevated risk in females in the age group 20-29 years (14).

Previous studies have suggested that adjustment disorders are more commonly found in women than men (11, 21). Additionally, evidence suggests that at the pretreatment stage 16% of infertile couples and only 2% of fertile couples have significant adjustment problems. The clinical and subclinical features of emotional distress may be present in the couples during their 1st visit to the infertility expert (22). Mood disorders (depression and dysthymia) are prevalent in patients with infertility (23- 25). These disorders are likely to worsen within the first three years of diagnosis and treatment (24-26). The latter sources also report that women are at a high risk for the emergence of reproductive mood disorders during the early child-bearing years. Researchers in Indian setups have also indicated that the prevalence of major depressive disorder is higher in women than in men. Men on the other hand are more often found to have mixed affective disorders (14, 15, 26-28). Depression is predicted by age, cause and duration of infertility, education, occupation and coping styles (8, 27, 29).

fertile couples have significant adjustment problems. The clinical and subclinical features of emotional distress may be present in the couples during their 1st visit to the infertility expert (22). Mood disorders (depression and dysthymia) are prevalent in patients with infertility (23- 25). These disorders are likely to worsen within the first three years of diagnosis and treatment (24-26). The latter sources also report that women are at a high risk for the emergence of reproductive mood disorders during the early child-bearing years. Researchers in Indian setups have also indicated that the prevalence of major depressive disorder is higher in women than in men. Men on the other hand are more often found to have mixed affective disorders (14, 15, 26-28). Depression is predicted by age, cause and duration of infertility, education, occupation and coping styles (8, 27, 29).

This research was planned knowing that: i. In India the rates of depression and anxiety rise in women above the age of 18 years, ii. Both these disorders contribute to considerable disease burdens and disability in work, family life and social functioning, and iii. There is a lack of empirical investigations estimating the prevalence of psychiatric disorders "in infertile women" in the Indian sub-continent. In this context, the aim of this study is to estimate the prevalence of psychiatric disorders in women seeking fertility treatments in a clinic based in Southern India.

The study is cross sectional and uses a convenience sample which is a part of a larger investigation of predictors of distress in infertile women (15). Sample size calculations were based on this larger investigation which included 300 Kannada/English/Hindi speaking women who were married, aged 22-50 years, had been diagnosed with primary infertility, and were seeking fertility treatments at Manipal Assisted Reproduction Centre (MARC), Kasturba Medical College, Manipal Academy of Higher Education (MAHE), Manipal. The study included all the women and their accompanying relative (spouse/family member) who consented to participate, and excluded women with secondary infertility and those unwilling to participate. Institutional Ethical clearance was taken with IEC number 275/2014.

The study is cross sectional and uses a convenience sample which is a part of a larger investigation of predictors of distress in infertile women (15). Sample size calculations were based on this larger investigation which included 300 Kannada/English/Hindi speaking women who were married, aged 22-50 years, had been diagnosed with primary infertility, and were seeking fertility treatments at Manipal Assisted Reproduction Centre (MARC), Kasturba Medical College, Manipal Academy of Higher Education (MAHE), Manipal. The study included all the women and their accompanying relative (spouse/family member) who consented to participate, and excluded women with secondary infertility and those unwilling to participate. Institutional Ethical clearance was taken with IEC number 275/2014.

The data in this study was collected using the following study tools. A brief form was compiled by the researchers for assessing socio-demographic variables. The second tool was the World Health Organization ‘International Classification of Diseases-Clinical Descriptive and Diagnostic Guidelines, 10th revision’ (ICD-10) (30). For the study, each woman’s history of psychological problems/psychiatric disorders was collected during a detailed psychological consultation. It was conducted by the principal investigator, a licensed Clinical Psychologist trained in the use of ICD. The psychiatric history provided by the participants was corroborated for reliability and validity with the accompanying relative.

Data collection in this study involved the following steps. After ethical clearance, participants were enrolled on the basis that they met the inclusion criteria. The purpose of the study and its implications were explained, together with the participants’ right to complete confidentiality and their right to withdraw from the study. Informed consent was taken from all of the women and their accompanying relatives who were willing to take part. Thereafter, the women completed the structured interview for the assessment of relevant socio-demographic and clinical variables. The assessment of the presence of psychiatric disorder was done using the ICD-10. Those found to have a significant psychiatric disorder were psycho-educated on their levels of distress and given the option of a consultation in the Department of Psychiatry and given a referral for the same.

All statistical analyses are carried out using SPSS version 15.0 (SPSS Inc., Chicago, IL, USA).

Figure 1 presents the frequency counts and percentage of women with and without mental health problems. Table 1 presents details of various psychiatric disorders prevalent in the participants.

**Fig 1:**
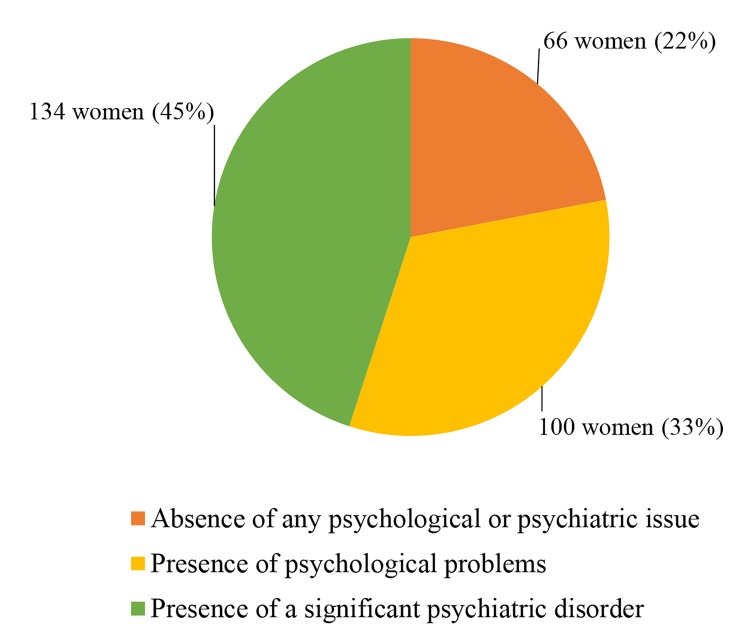
Frequency counts and percentage of women detected with and without mental health problems.

**Table 1:** Details of various psychiatric disorders prevalent in the participants


Details of various psychiatric disorders in infertile women (n=134)	Frequency data n (%)

i. Adjustment disorder (ICD 10 code F43.2)	49 (16)
ii. Anxiety NOS (ICD 10 code F41.9)	29 (10)
iii. Mixed anxiety and depressive disorder (ICD 10 code F41.2)	26 (9)
iv. Dysthymic disorder (ICD 10 code F41.2)	15 (5)
v. Major depressive disorder (ICD code 10 F 32)	12(4)
vi. Other anxiety disorders (social phobia, generalized anxiety disorder, obsessive compulsive disorder) (ICD 10 codes F40, F41.1, F42 respectively)	3 (1)


ICD; International classification of diseases and NOS; Not otherwise specified.

Results of this study reveal that the criterion for significant psychiatric disorder is met by 45% (134 out of 300) of the participants. This is followed by the ‘off and on’ presence of psychological problems reported by 33% (100 out of 300) of the women who partially met the criteria for an ICD-10 diagnosis of disorder, but did not meet the time duration requirement). Lastly 22% (66 out of 300) of the women are found to be free from any psychological or psychiatric problem.

Based on the current data serious psychopathology is found to be quite widespread in infertility. Additionally, a sizeable proportion of women suffer from sub-threshold symptoms of anxiety and depression, although they did not fulfil the minimum duration for any specific psychiatric disorder in ICD-10. The present findings are somewhat similar to recent research which showed that 54% (27 out of 50) of infertile females have a significant psychiatric disorder (14). However, international estimates reveal a higher prevalence rate (6, 11-13).

The most common psychiatric diagnosis established in the present research is Adjustment Disorder with mixed affective features (found in 49 out of 300, roughly 16% of women). This is followed by anxiety disorder (unspecified) reported by nearly 10% (29 out of 134) of the participants and lastly mixed anxiety and depressive disorder reported by around 9% (26 out of 300) of women. Other psychiatric conditions, such as social phobia, generalized anxiety disorder and obsessive compulsive disorder were reported by 1% (3 out of 300) of the women. These findings are similar to those observed by other researchers who have also shown that adjustment disorders, anxiety disorders and mood disorders are frequently manifested in infertile women (11, 14, 23, 31-33). Additionally, the literature highlights the association between unmanageable infertility distress and occurrence of complicated grief reactions, depression, dysthymia, reproductive mood disorders, anxiety disorders, adjustment disorders, as well as sexual dysfunctions in patients undergoing intra uterine insemination (IUI) and *in vitro* fertilization (IVF). All of these are known to comprise quality of life in infertile men and women (18-25). Studies also reveal that ‘anxiety features’ are co-morbidly present with infertility distress, and depression is found in more than 50% of childless patients (11, 27, 28). Furthermore, less than 15% of participants in this study reported having consulted a professional mental health practitioner. Statistics from other nations also depict a similar trend (23). The present data is concordant with recent studies suggesting that psychiatric issues are often overlooked and undiagnosed in a majority of infertile patients in India (10, 15, 26).

Dysthymic features are reported by patients in the present study. Yet, severe depressive features, suicidal ideations or hopelessness are not reported. The results of this study are similar to other studies demonstrating that mild depression is more prevalent than moderate or severe depression, particularly in Indian contexts (10, 28).

This study has certain limitations. Firstly, is the lack of separate assessments of the husbands of the women who participated in this research. Secondly, an unstructured psychiatric interview schedule was included as a measure to tap psychiatric disorders in this study. This could have been supplemented with a structured tool for increasing the diagnostic validity of presence of a specific psychiatric disorder. Thirdly, reporting/recall biases of the participants could have crept in our data. Further studies conducted on this subject may consider drawing comparisons between mental health issues in i. Infertile women in comparison to fertile controls, ii. In women who conceive with treatments versus those who do not, and iii. In women who remain childless versus whose who go on to adopt. Additionally, psychiatric/psychological disorder is known to be associated with variables like age, gender, occupation, treatment type, length and history, duration of infertility, costs of evaluation or cure and other psychosocial variables (11, 12, 32, 33). Thus, the predictors and protective factors for psychiatric disorders in women seeking treatment for infertility can also be established in prospective investigations.

Over the past fifty years, most countries have come up with evidenced based committee reports on assessing the psychological endurance of couples prior to commencement of infertility treatments as well as protecting their overall wellbeing at all stages of the treatment process.

In conclusion, our data reveals that the prevalence of psychiatric disorders is high in infertile women. Ensuring the emotional wellbeing of patients seeking fertility treatments in India is an important component of comprehensive clinical care.
